# Targeting Epigenetic Regulatory Enzymes for Cancer Therapeutics: Novel Small-Molecule Epidrug Development

**DOI:** 10.3389/fonc.2022.848221

**Published:** 2022-03-28

**Authors:** Ye Jin, Tianjia Liu, Haoming Luo, Yangyang Liu, Da Liu

**Affiliations:** ^1^ School of Pharmacy, Changchun University of Chinese Medicine, Changchun, China; ^2^ Affiliated Hospital of Changchun University of Chinese Medicine, Changchun, China

**Keywords:** epigenetic regulatory enzymes, cancer therapeutics, inhibitors, small molecule, epidrug development

## Abstract

Dysregulation of the epigenetic enzyme-mediated transcription of oncogenes or tumor suppressor genes is closely associated with the occurrence, progression, and prognosis of tumors. Based on the reversibility of epigenetic mechanisms, small-molecule compounds that target epigenetic regulation have become promising therapeutics. These compounds target epigenetic regulatory enzymes, including DNA methylases, histone modifiers (methylation and acetylation), enzymes that specifically recognize post-translational modifications, chromatin-remodeling enzymes, and post-transcriptional regulators. Few compounds have been used in clinical trials and exhibit certain therapeutic effects. Herein, we summarize the classification and therapeutic roles of compounds that target epigenetic regulatory enzymes in cancer treatment. Finally, we highlight how the natural compounds berberine and ginsenosides can target epigenetic regulatory enzymes to treat cancer.

## 1 Introduction

The concept of epigenetics was first introduced in 1942 by Waddington, a British scientist who defined “epigenetics” as changes in the phenotype without underlying genotypic changes to explain altered growth and development (1). Epigenetics is now widely recognized as the regulatory mechanisms by which a heritable phenotype is changed without altering the DNA sequence. Epigenetic changes, including DNA/RNA methylation, histone modifications, nucleosome localization, non-coding RNA (ncRNA) expression, and chromatin 3D structure, are involved in cellular growth, development, and function (2). These epigenetic modifications constitute the specific epigenome of an individual organism and provide a regulatory mechanism for cellular diversity. Recently, epigenetics has gained attention in fields such as medicine, exerting a profound impact on the research and treatment of diseases such as cancer.

Epigenetic modifications catalyzed by epigenetic regulatory enzymes are important for regulating chromatin structure and gene expression. Imbalanced gene expression can be one of the main mechanisms underlying diseases such as cancer. In particular, thee aberrant expression of oncogenes, tumor suppressor genes, or cancer-related genes by dysregulated epigenetic regulatory enzymes can trigger tumorigenesis by modulating basic processes, such as DNA repair, cell proliferation, and mortality (3, 4). Therefore, epigenetic marks such as DNA methylation, histone modifications, and ncRNA expression have been identified as potential biomarkers for the early diagnosis and prognosis of cancers (5, 6). In recent years, many small-molecule compounds targeting epigenetic regulatory enzymes have been discovered, some of which are promising anticancer drugs.

The discovery and development of inhibitors targeting epigenetic regulatory enzymes are extensively described in this review. Further, we summarize the functions of berberine (BBR) and ginsenosides, natural compounds capable of targeting epigenetic enzymes in cancer. Additionally, we discuss promises and challenges that lie ahead of us.

## 2 DNA Methylation and Its Role in Cancer Treatment

### 2.1 DNA Methylation

**Figure 1:**
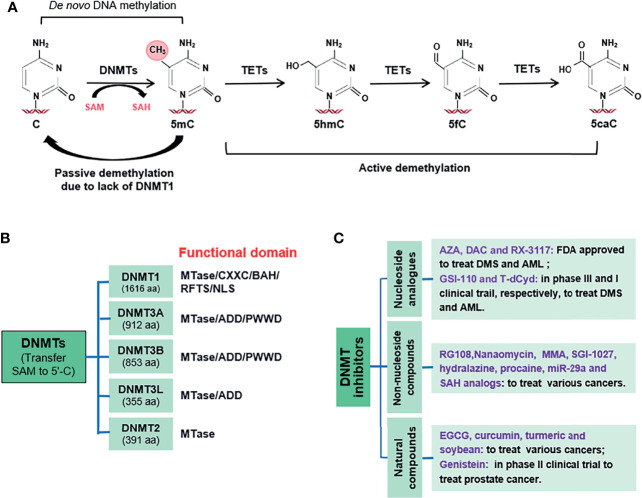
Types of DNA methyltransferases (DNMTs) and antitumor activity of its inhibitors: **(A)**. Schematic of DNA methyltransferases (DNMTs) transferring methyl donor S-adenosyl-L-methionine (SAM) to cytosine (5 ‘C) 5’- residues in DNA or removing SAM from DNA. **(B)**. Functional domains of DNMTs. (DNMT1, DNMT2, DNMT3A, DNMT3B and DNMT3L) **(C)**. Three types of DNMTs inhibitors.

DNA methylation is a stable epigenetic event in intracellular processes, such as cell differentiation, and is involved in the lineage classification and quality control of stem cells (7). In humans, DNA methylation occurs almost exclusively at cytosine residues in CpG sequences. These dinucleotides are dispersed unevenly across the genome, and most are heavily methylated. In contrast, CpG-rich regions known as CpG islands (CpGIs) remain largely unmethylated, especially in promoter regions (8). However, altered CpGI methylation patterns during cancer progression result in both genome-wide hypomethylation and site-specific CpGI hypermethylation (9). Therefore, DNA methylation provides a useful molecular marker for cancer diagnosis and therapeutics (10). In mammals, DNA methyltransferases (DNMTs) are responsible for transferring methyl donor S-adenosyl-L-methionine (SAM) to the 5′-residue of cytosine (5′-C) in DNA (**Figure 1A**). The DNMT family includes DNMT1, DNMT2, DNMT3A, DNMT3B, and DNMT3L, which differ based on their structural characteristics and functional domains (**Figure 1B**). For example, the Pro-Trp-Trp-Pro (PWWP) domain of DNMT3A/3B recognizes the di- or tri-methylation of histone H3 lysine 36 (H3K36) to activate gene expression (11, 12), whereas the ATRX-like domain of DNMT3A and XXC-BAH1 domain of DNMT1 interact with deacetylase HDAC1 to repress gene expression (13, 14). Further, the C-terminal catalytic methyltransferase (MTase) domain of DNMT3A mediates homo- and heterodimerization to regulate progressive DNA methylation (15, 16). Cleavage between the N- and C-terminal domains reportedly affects the relative preference of DNMTs for unmethylated and hemi-methylated DNA (17). DNMTs preferentially bind to hemi-methylated CpG sites (18).

DNA demethylation can occur either passively or actively. DNA demethylation or “erasing DNA methylation” can occur passively when DNA is replicated and the modification is not re-established. One example of passive DNA demethylation is the absence of methylation owing to a lack of DNMT1, whereas another is the removal of methyl groups from cytosine (5-mC) by ten-eleven translocation proteins (TETs) in a replication-independent manner. TETs mainly oxidize 5-mC to form 5-hydroxymethylcytosine (5hmC), 5-formylcytosine (5fC), and 5-carboxycytosine (5caC) (19, 20). The catalytic domain of TETs consists of a double-stranded β-helix domain and cysteine-rich domain at the carboxyl end (21). In addition, TET1 and TET3 contain CXXC domains at their N-terminus, which are composed of two Cys4-type zinc finger motifs that promote DNA binding (22). Importantly, DNA methylation can be recognized by methyl-CpG-binding proteins (MBPs), which bind and interpret methylated DNA to initiate gene silencing by recruiting other factors (23). MBPs can be classified as methyl-CpG-binding domain (MBD) proteins as follows: su(var) 3-9, enhancer of zeste, and trithorax (SET), RING-associated, and zinc finger (24). To date, 11 proteins in the MBD family have been identified, including methyl-CpG-binding protein 2, MBDs 1–6, SETB1/2, and BAZ2A/B (25). In addition to the MBD domain, SETB1/2 and BAZ2A/B also contain other domains, such as SET, DNA-binding homeobox and different transcription factors, plant homeodomain (PHD), and bromodomain (BRD). Although they cannot interact with 5mC residues, MBPs can bind to methylated or acetylated histones to participate in heterochromatin formation and transcriptional inhibition by coordinating H3K9 demethylation, histone H4 deacetylation, and DNA methylation, which are essential for the epigenetic silencing of ribosomal DNA (26, 27).

### 2.2 Inhibitors Targeting DNMTs (DNMTis)

In view of the hypermethylation of CpGIs in the promoter region of most cancers, DNMT inhibitors (DNMTis) have been developed for tumor treatment. DNMTis are mainly divided into three types, nucleoside analogs (NAs), non-nucleoside compounds, and natural compounds (**Figure 1C**) (28). Compounds that inhibit DNMTs lead to hypomethylation across cell divisions, subsequently inducing the expression of tumor suppressors. Using methylation-specific PCR, Chan et al. demonstrated substantial demethylation of all latent and lytic Epstein-Barr virus promoters in nasopharyngeal cancer patients after treatment with 5-azacytidine (a DNMTi) (29). DNMTis such as azacitidine, decitabine, guadecitabine, and 4-thio-2-deoxycytidine have been examined in clinical anti-tumor trials (30–32) (**Table 1**). Non-nucleoside compounds with various chemical scaffolds have also been studied (63). Compounds such as RG108, nanaomycin A, mithramycin A, SGI-1027, hydralazine, procaine, S-adenosyl-L-homocysteine analogs, and miR-29a have been shown to suppress the activity of DNMTs (33–40). Among these, hydralazine has been shown to be an effective demethylation agent and tumor suppressor gene transcriptional reactivator (36). In a phase II clinical study, hydralazine in combination with standard cytotoxic chemotherapy reactivated tumor suppressor genes silenced by DNA methylation and increased chemotherapy efficacy in prostate cancer (33). Interestingly, some natural compounds, such as (-)-epigallocatechin-3-gallate, curcumin, and genistein from green tea/soybean, also reportedly block the activity of DNMTs (36, 37). Genistein and related soy isoflavones reportedly reactivate methylation-silenced genes to delay the progression of breast or prostate cancer by directly blocking DNMT. Although many DNMTis have been identified, few have been applied clinically as current DNMTis are nonselective cytosine analogs that induce cytotoxic side effects (64).

**Table 1:** Small molecule compounds targeting epigenetic regulatory enzymes.

Compound	Type	Tumor types	Status	Ref.
DNMTi				
5-azacytidine/AZA	NA	DMS/AML	Phase I	(29)
5-aza-2’deoxycytidine/DAC	NA	DMS/AML	Phase I	(29)
RX-3117	NA	DMS/AML	Phase I	(30)
Guadecitabine/SGI-110	NA	AML	Phase II	(31)
4-Thio-2-deoxycytidine	NA	Cancer	N/A	(32)
RG108	NNC	Prostate cancer	N/A	(33)
Nanaomycin A	NNC	Colorectal cancer	Phase III	(33)
Mithramycin A/MMA	NNC	Lung cancer	N/A	(33)
SGI-1027	NNC	Cancer	N/A	(34)
Procaine	NNC	Human cancer	N/A	(35)
Hydralazine	NNC	Prostate cancer	Phase I	(36)
SAH analogs	NNC	MDS	N/A	(37)
MiR-29a	NNC	AML	N/A	(38)
EGCG	Natural compounds	Colon Cancer	Phase I	(39)
Curcumin/Genistein	Natural compounds	Breast Cancer	Phase II	(39)
Soybean	Natural compounds	Prostate Cancer	Phase II	(40)
HMTi				
BIX-01294	G9a-GLP inhibitors	Prostate/colon cancer	N/A	(41)
Chaetocin	Non-specific inhibitor	Glioma cancer	N/A	(41)
GSK343	LIS	Osteosarcoma	CTT	(42)
CPI-1205/UNC0321	LIS	Solid tumors/BCL	Phase I	(43)
UNC1999	LIS	Bladder cancer	CTT	(25)
EPZ005687/GSK-126/EL	LIS	DLBL	CTT	(25)
Tazemetostat/EPZ6438	LIS	Solid tumors/BCL	Phase I	(44)
Tazemetostat	LIS	follicular lymphoma	Phase 2	(45)
Tazemetostat	LIS	Papillary thyroid cancer	N/A	(46)
EPZ004777	DOT1L inhibitor	Leukemia	N/A	(47)
EPZ-5676	DOT1L inhibitor	Leukemia	Phase I	(48)
SYC-522	DOT1L inhibitor	AML	N/A	(49)
PRMTi				
DB75	Type I PRMT Inhibitor	Malaria	Phase I	(44)
GSK3368715	Type I PRMT Inhibitor	Solid tumors	Phase I	(50)
TP-064/EZM2302	CARM1 inhibitor	MM	N/A	(51)
GSK3235025/EPZ015666	PRMT5 inhibitor	NHL	N/A	(52)
GSK3326595/EPZ015938	PRMT5 inhibitor	Breast cancer	Phase II	(52)
Ly -283	PRMT5 inhibitor	NHL	N/A	(53)
GSK3203591	PRMT5 inhibitor	Breast cancer	N/A	(54)
KDMi				
PCPA	LSD1 inhibitor	Cancer	N/A	(55)
INCB059872	LSD1 inhibitor	Myeloid leukemia	Phase I	(56)
IMG-7289	LSD1 inhibitor	Acute myeloid leukemia	Phase I	(57)
CC-90011	LSD1 inhibitor	Prostatic cancer	Phase I/II	(58)
Thieno[3,2-b]pyrrole-5-carboxamides	LSD1 inhibitor	Human leukemia	N/A	(59)
GSK2879552	PCPA derivatives	AML/SCLC	Phase I	(60)
ORY-1001	PCPA derivatives	AML/SCLC	Phase I	(61)
HCI-2059	PCPA derivatives	MYCN-amplified neuroblastoma	N/A	(62)

AML, Acute Myeloid Leukemia; BCL, B-cell lymphoma; CTT, Clinical trial termination; DLBL, Diffuse large B-cell lymphoma; EGCG, (-)-epigallocatechin-3-gallate; LIS, Lyridine-indazole scaffold; MDS, Myelodysplastic syndrome; MM, Multiple myeloma; NA, Nucleoside analogues; NHL, Non-Hodgkin’s lymphoma; NNC, Non-nucleoside compounds; SCLC, Small cell lung cancer.

## 3 Histone Methylation as an Anticancer Target

### 3.1 Histone Methylation

**Figure 2:**
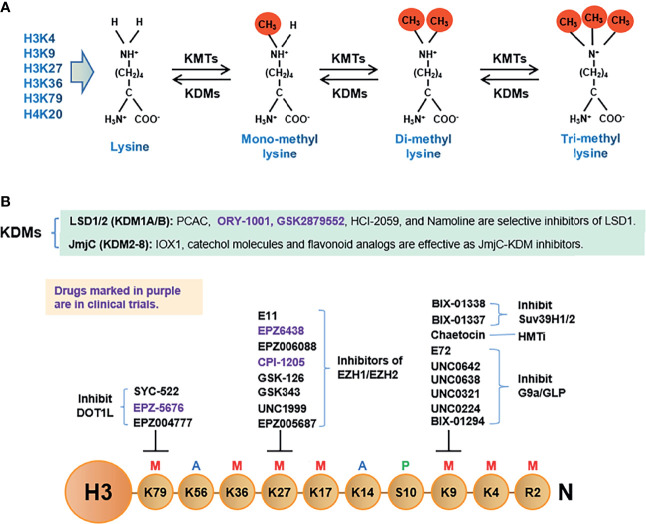
The histone methylation process, inhibitors of histone lysine methyltransferases (HMTs) and histone lysine demethylases (KDMs). **(A)**. Schematic diagram of three states of lysine methylation: mono, di, and tri states. **(B)**. A summary of the histone methylation process and inhibitors of HMTs and KDMs.

Histone methylation, a unique post-translational modification catalyzed by histone MTases (HMTs), occurs at both lysine (K) and arginine (R) residues. Abnormal histone methyl modification plays an important role in the proliferation, apoptosis, differentiation, and invasion of tumor cells. Thus, blocking these abnormal modifications has become a new direction in tumor therapeutics (65, 66). The key steps of the histone methylation process, including HMT inhibitors (HMTis) and histone lysine demethylases (KDMs), are shown in **Figures 2A, B**. Lysine methylation occurs in mono-, di-, and tri-states, whereas arginine methylation only occurs in mono-and di-states. These methyl marks contribute to transcriptional regulation and serve as platforms for the recruitment of effector proteins. Most HMTs contain the SET domain. Methylation occurs at lysine residues K4, K9, K27, K36, and K79 of histone H3 and K20 of histone H4 (**Figure 2B**). In general, methylation at H3K9, H3K27, and H4K20 correlates with transcriptional repression, whereas methylation at H3K4, H3K36, and H3K79 corresponds with gene transcription (67). H3K9me2/me3, H3K27me2/me3, and H4K20me3 often appear on heterochromatin where genes remain silent (68).

There are two families of histone demethylases, lysine-specific demethylases (LSDs) and Jumonji C (JmjC) domain-containing lysine demethylases (JmjC-KDMs). The LSD family includes LSD1/KDM1A and LSD2/KDM1B proteins, which contain the N-terminal Swi3p, Rsc8p, and Moira (SWIRM) domains, a flavin adenine dinucleotide-binding motif (FAD), and a C-terminal amine oxidase domain that is responsible for LSD activity in an FAD-dependent manner (69). Both LSD1 and LSD2 function as corepressors through the demethylation of mono- or di-methyl marks on H3K4 (70). However, LSD1 can also act as a coactivator of the androgen receptor *via* the demethylation of H3K9me1/me2 (71). The JmjC-KDM family includes iron- and α-ketoglutarate-dependent dioxygenases, which can be divided into KDM 2–8 subfamilies. Members of the JmjC-KDM family are responsible for the demethylation of all statuses of H3K4, H3K9, H3K27, H3K36, H3K79, and H4K20 through the co-substrate 2-oxoglutarate, dioxygen, and Fe (II) as a cofactor (42, 72). The lysine residues mentioned previously herein are prone to methylation and play critical roles in tumorigenesis (73, 74).

### 3.2 Histone Methyltransferase Inhibitors (HMTis)

Histone methylation is a hot topic in tumor epigenetic modification. This modification is associated with the biological behavior of tumor cells and plays a role in the development of tumors. In this section, we focus on a subclass of epigenetic regulators, namely histone methyltransferases. To date, hundreds of HMTs have been identified, including lysine and arginine MTases (47). Several HMTs have been linked to different types of cancer. However, in most cases, we only have limited knowledge regarding the molecular mechanisms by which the HMTs contribute to disease development. HMTis can be classified according to their specificity for different types of methyltransferases. Here, we summarize the current knowledge regarding some of the best validated examples of HMTis inhibiting tumorigenesis and discuss their potential mechanisms of action.

#### 3.2.1 Inhibitors of H3K9 HMTs

Most HMTs are present as closely homologous pairs. For example, the catalytic SET domains of G9a and GLP share 77% sequence identity and are present as a stoichiometric heterometric complex (75). In cells, they are responsible for H3K9 methylation and G9a/GLP-mediated H3K9me2, which are highly associated with transcriptional repression (76). A recent study reported high expression of G9a in various cancers, such as prostate/colon/lung cancers, multiple myeloma, and lymphocytic leukemia, indicating that G9a inhibitors might suppress cancer proliferation (41, 77). BIX-01294 was first found as a G9a/GLP-specific inhibitor that can modulate global H3K9me2 levels in cells (78). Although specific G9a/GLP inhibitors, such as UNC0224, UNC0321, E72, UNC0638, and UNC0642, have been developed, they have not been used in clinical trials because of their cell toxicity or poor bioavailability.

#### 3.2.2 Inhibitors of H3K27 HMTs

In mammals, polycomb repressive complex 2 exhibits HMT activity on H3K27 *via* catalytic subunits enhancer of zeste homologs 1/2 (EZH1/2) (79). In *Drosophila*, EZH1 and 2 are mainly responsible for maintaining the spatial expression pattern of homeobox (*HOX*) genes (80). Aberrant EZH2 expression has been associated with various human cancers. For example, the overexpression of EZH2 has been detected in prostate, breast, and other cancers, suggesting that it might serve as a prognostic marker for cancers (43, 81). Further, wild-type and mutant EZH2 cooperatively regulate and maintain the hypertrimethylation of H3K27, which inhibits the proliferation of lymphoma cells by abnormally silencing PCR2-target genes (47).

Inhibitors of EZH1/2 can be classified into three groups according to their basic skeleton structure as follows: those with the pyridone-indazole scaffold, which includes EPZ005687, UNC1999, and GSK343 (48); those with the pyridone-indole scaffold, which includes GSK-126, CPI-1205, and E11 (41, 78); and those with the pyridone-phenyl scaffold, which includes EPZ006088 and EPZ6438 (tazemetostat) (45). More recently, several non-SAM-derived inhibitors of the catalytic activity of EZH2 have been discovered. Among them are GSK126 and EPZ005687, inhibitors effective against EZH2 mutant lymphomas, and EI1, a low MW inhibitor that blocks diffuse large B-cell lymphoma proliferation (46). Tazemetostat has been recently approved for relapsed/refractory after two or more lines of therapy in the presence of an EZH2 mutation or independent of an EZH2 mutation in the absence of other options (82). Combined tazemetostat and MAPKis enhances the differentiation of papillary thyroid cancer cells harboring BRAFV600E by synergistically decreasing the global trimethylation of H3K27me (44). UNC1999, a modified inhibitor, improves the specificity of EZH2 and achieves better oral bioavailability (83). As a second-generation compound, EPZ6438 shows improved potency, pharmacokinetic properties, and selectivity for EZH1 than EPZ005687 (81). Both EPZ6438 and CPI-1205 are currently undergoing clinical trials for solid tumors or B-cell lymphoma (84) (**Table 1**).

#### 3.2.3 Inhibitors of H3K79 HMTs

DOT1-like protein (DOT1L), an enzyme responsible for H3K79 methylation, does not contain the SET catalytic domain and displays a class I SAM-dependent MTase fold (85). In cells, DOT1/DOT1L-mediated H3K79 methylation is involved in various biological processes, including gene transcription, the cell cycle, and DNA damage repair (86). DOT1L interacts with mixed lineage leukemia (MLL) translocation fusion proteins, such as AF10, ENL, AF9, and AF4, resulting in the DOT1L-mediated H3K79 methylation of target genes. Therefore, DOT1L has become a potential target for developing therapeutic drugs to treat leukemia.

To date, more than 20 DOT1L inhibitors have been reported. Among them, EPZ004777 was first found to selectively kill leukemic cells by repressing DOT1L-mediated H3K79 methylation (87). EPZ-5676, an optimized version of EPZ004777, forms hydrogen bonds with residues Asp222, Glu186, Gly163, and Asp161 of DOT1L to prevent cellular H3K79 methylation. EPZ-5676 has been used against leukemia in phase I clinical trials (88). Another DOT1L inhibitor, SYC-522, effectively delayed the progression of MLL in the preclinical phase by suppressing H3K79 methylation and reducing the expression of two important leukemia-related genes, *HOXA9* and *MEIS1*. Additionally, SYC-522 significantly reduces the expression of *CCND1* and *BCL2L1*, which are important regulators of the cell cycle and anti-apoptotic signaling pathways (49) (**Table 1**).

### 3.3 Inhibitors Targeting Protein Arginine Methyltransferases (PRMTis)

The protein arginine MTase (PRMT) family includes nine enzymes divided into three types, type I PRMT, CARM1, and PRMT5. In cells, PRMTs catalyze the methylation of arginine residues on histones. PRMT dysfunction is associated with the occurrence of several cancers.

PRMTis are also classified into three types based on their corresponding PRMT type and have been investigated in the early preclinical stage (**Table 1**). Type I PRMTis include AMI-1, AMI-6, DB75, GSK3368715, and MS023 (50, 89). MS049, TP-064, and EZM2302 exhibit the highly selective inhibition of CARM1 (PRMT4) (51), and the latter two compounds can be effectively used to treat multiple myeloma (MM) by selectively blocking CARM1 (90, 91). Interestingly, PRMT5 inhibitors, such as EPZ015666 (GSK3235025), EPZ015938 (GSK3326595), and LLY-283, possess high anti-tumor activities. EPZ015666 was used against NHL in clinical trials by blocking SmD3 methylation (52–54).

### 3.4 Inhibitors Targeting Histone Lysine Demethyltransferases (KDMis)

Many small-molecule compounds have emerged as lysine demethyltransferase inhibitors (KDMis), some of which have entered different clinical stages as anti-tumor drug candidates (**Table 1**). Inhibitors of both the LSD/KDM and JmjC-KDM family proteins have been shown to block the catalytic domain to reduce catalytic activity. One of the most potent LSD1 inhibitors, tranylcypromine (PCPA), causes the irreversible inhibition of LSD1 by forming a covalent adduct with the FAD cofactor of LSD1 (92). This process destroys the catalytic group of the histone lysine demethyltransferase, which inhibits the activity of the enzyme and inactivates it. Based on the chemo-type scaffold, a series of PCPA derivatives have been designed and shown to exert anti-tumor effects (55). Two recently developed PCPA derivatives, ORY-1001 and GSK2879552, promoted the differentiation of acute myeloid leukemia (AML) and limited the growth of small cell lung cancer (SCLC) in a phase 1 clinical trial aimed to assess their roles against AML and SCLC (60, 61). These two PCPA derivatives exhibit higher selectivity for LSD1 than for PCPA (93). Therefore, PCPA derivatives have the potential to become new epigenetic anticancer drugs. In addition, HCI-2509, a potent small-molecule inhibitor of LSD1, hinders the growth of and exerts the cytotoxic effects on neuroblastoma (NB) cells *via* p53 (62). Of the thieno[3,2-b]pyrrole-5-carboxamides, novel reversible inhibitors of KDM1A, that showed a remarkable anti-clonogenic cell growth effect on MLL-AF9 human leukemia cells (59) (**Table 1**).

Various structural scaffolds, including hydroxamic acid, hydroxyquinoline analogs, and cyclic peptides, reportedly function as effective JmjC-KDM inhibitors (25). For example, the 8-hydroxyquinoline derivative IOX1 can block many KDM isoforms (94). Several catechol molecules and flavonoid analogs have also been identified as JmjC-KDM inhibitors (25). However, the aforementioned compounds are still in the developmental phase.

### 3.5 Inhibitors Targeting Specific Functional Domains of Methyl-Lysine Readers

The methylation of lysine residues in N-terminal tails of histones H3 and H4 widely mediates biological processes in cells. In recent decades, various proteins containing specific functional domains that recognize methyl-lysine on histones have been identified, such as methyl-lysine reader proteins. Methyl-lysine readers are approximately categorized into chromodomain, PHD finger domain, Tudor domain, PWWP domain, WD40 repeat (WDR) domain, and malignant brain tumor (MBT) domain families (5). These proteins exhibit different abilities to recognize methylated lysine residues according to their different functional domains.

Chromodomain proteins are further classified into heterochromatin protein 1 (HP1)/polycomb (Pc), chromo-ATPase/helicase-DNA-binding (CHD), chromobarrel domain, and chromodomain Y (CDY) families (5). Both HP1/Pc and CDY proteins show strong preference for trimethylated H3K9 and H3K27 (68). Moreover, CHD proteins recognize methyl-lysine residues on H3K4 (95), whereas chromobarrel domain proteins interact with methylated H3K36 and H4K20 (96). In addition, both PHD and MBT domain proteins recognize methylated H3K4 (97).

Methyl-lysine reader proteins play important roles in regulating many cellular processes, such as development, the cell cycle, stress responses, and oncogenesis, and have increasingly become the focus of epigenetic research. Inhibitors of methyl-lysine reader proteins, such as MS37452A, SW2_110A, and UCN3866, have been found to inhibit the growth of cancer cells as selective inhibitors of Pc chromobox (CBX) and CDY proteins (98, 99). Additionally, several compounds have been identified as PHD inhibitors (100). For example, macrocyclic calixarenes can disrupt the binding of ING2 PHD to H3K4me, disulfiram, amiodarone, and tegaserod to prevent interactions between JARID1A PHD3 and H3K4me3 (2, 6). Moreover, benzimidazole can be selectively docked in methylated H3K4, preventing the binding of the Pygo-BCL9 chromatin reader to H3K4me PHD (101). Thus, many proteins targeting methyl-lysine readers have been shown to exert anticancer effects (102).

## 4 Histone Acetylation as a Target for Anti-Tumor Drug Development

### 4.1 Histone Acetylation

Acetylation of the ε-amino group of a lysine residue was first discovered with histones in 1968, but the responsible enzymes, histone acetyltransferases and deacetylases, were not identified until the mid-1990s (103). Histone acetylation is a reversible process that occurs *via* the addition of an acetyl group to the ε-amino of the lysine residue at the midamino end and tail of the histone. This process is dynamically controlled by histone acetyltransferases (HATs), lysine acetyltransferases, and histone deacetylases (HDACs) (**Table 2**). Lysine residues on histones are prone to acetylation, resulting in a decrease in the positive charge and weakening of the interaction between histones and DNA (104).

**Table 2:** Classification of histone deacetylases (HDACs) and their inhibitors.

Classification			Locations	Inhibitors
Zn++ Dependent	Class I	HDAC1 HDAC2 HDAC3 HDAC8	Nucleus Nucleus Nucleus/cytoplasm Cytoplasm	pan-HDAC inhibitors approved by FDA to treat CTCL, PTCL, AML: Vorinostat (SAHA), Belinostat (PXD-101), Panobinostat (LBH589), Pracinostat (MEI pharma), Romidepsin (FK228) Chidamide (CS055, HBI-8000) pan-HDAC inhibitors are being evaluated clinically: Resminostat (4SC-201) →for Hodgkin’s lymphoma; Givinostat (ITF2357) →for polycythemia; Quisinostat (JNJ-26481585), Entinostat and Mocetinostat →for various cancers. pan-HDAC inhibitor in preclinical stage: Trigustatin A
	Class IIa	HDAC4 HDAC5 HDAC7 HDAC9	Cytoplasm/nucleus Cytoplasm/nucleus Cyto–/mto-/nucleus Cytoplasm/nucleus	
	Class IIb	HDAC6 HDAC10	Cytoplasm Cytoplasm/nucleus	
	Class IV	HDAC11	Nucleus	
NAD+ Dependent	Class III	SIRT 1 SIRT 2 SIRT 3 SIRT 4 SIRT 5 SIRT 6 SIRT 7	Cytoplasm Cytoplasm/nucleus Mitocondria Mitocondria Mitocondria Nucleus Nucleus	SIRTs inhibitors for against breast cancer: Sirtinol and Nicotinamide

There are three major families of HATs, general control non-derepressible 5 (Gcn5)-related N-acetyltransferases (GNATs), p300/CBP, and MYST proteins. p300 (adenoviral E1A-associated protein of 300 kDa) and CBP (CREB-binding protein) form a pair of paralogous transcriptional co-activators. Members of the GNAT family include HAT1, yeast Gcn5, and its metazoan orthologs GCN5 and PCAF (p300/CBP-associated factor) (103). HATs are classified into types A and B based on their cellular location. Type A is responsible for acetylating histones associated with chromatin, whereas type B acetylates newly translated histones in the cytoplasm. Nuclear HATs can be divided into two categories based on their sequence homology and shared structural features. The GCN5-related N-acetyltransferase (GNAT) family, which includes GCN5 and p300/CBP-associating factor (PCAF), can acetylate lysine residues on histones H2B, H3, and H4. Meanwhile, the MOZ, YBF2/SAS3, SAS2, and TIP60 (MYST) families of proteins are characterized by a highly conserved MYST domain (105).

Acetyl groups on lysine residues must be removed by HDACs. Dependent on sequence similarity and cofactor dependency, HDACs are grouped into four classes and two families, the classical and silent information regulator 2 (Sir2)-related protein (sirtuin) families. In humans, members of the classical family include HDAC1, 2, 3, and 8 (class I); HDAC4, 5, 6, 7, 9, and 10 (class II); and HDAC11 (class IV). They share sequence similarity and require Zn^2+^ for deacetylase activity. The sirtuin family contains seven members (SIRT1–7, class III), which show no sequence resemblance to members of the classical family and require NAD+ as the cofactor (106, 107).

### 4.2 Inhibitors Targeting Histone Acetyltransferases (HATis)

Imbalanced HAT expression and acetylation levels in tumorigenesis make HATs suitable targets for drug development. In preclinical experiments, many small-molecule compounds have been screened as potential HATis to regulate histone acetylation and reduce tumor growth (**Table 3**). These compounds include isothiazolone-based chemical compounds, the natural compounds garcinol and embelin (108, 109), the pyrazolone-containing small molecule C646 (124), and the pyridoisothiazole derivatives PU139 and PU141, which block PCAF and/or p300 (110). For example, PU139 retards the growth of NB by blocking Gcn5, PCAF, CBP, and p300 (110).

**Table 3:** Compounds targeting histone acetylation exert anti-tumor activity.

Compound	Types	Tumor types	Status	Ref.
HATi				
Garcinol	Natural compound	Breast cancer	Preclinical	(108)
Embelin	Natural compound	Prostate cancer	Preclinical	(109)
PU139	A pyrazolone containing small molecule C646	Neuroblastoma	Preclinical	(110)
PU141	Pyridoisothiazole derivatives	Neuroblastoma	Preclinical	(110)
HDACi				
Trigustatin A	Zinc-dependent HDACs inhibitors	NA	Preclinical	(111)
Vorinostat/SAHA	P-HDACi	CTCL	Phase II	(112)
Belinostat/PXD-101	P-HDACi	PTCL	Approved by FDA	(113)
Panobinostat/LBH589	P-HDACi	MM	Approved by FDA	(114)
Pracinostat/MEI pharma	P-HDACi	AML	Phase II	(115)
Romidepsin/FK228	P-HDACi	CTCL	Phase II	(116)
Chidamide/CS055/HBI-8000	P-HDACi	AML	Phase I	(117)
Resminostat/4SC-201	P-HDACi	Solid tumors	Phase I	(118)
Givinostat/ITF2357	P-HDACi	Polycythemia	N/A	(119)
Quisinostat/JNJ-26481585	P-HDACi	Solid tumors	Phase I	(120)
Entinostat	P-HDACi	HL	Phase II	(121)
Mocetinostat	P-HDACi	HL	Phase II	(122)
Sirtinol/Nicotinamide	SIRTs inhibitors	Breast cancer	Phase I	(123)

CTCL, Cutaneous T-cell lymphoma; HL, Hodgkin lymphoma; MPNs, myeloproliferative neoplasms; P-HDACi, Pan-HDAC inhibitors; PTCL, peripheral T-cell lymphoma.

### 4.3 Inhibitors Targeting Histone Lysine Deacetylases (HDACis)

Global histone acetylation levels are frequently decreased in cancer cells. Correcting imbalanced acetylation in tumor cells can be achieved by reducing the activity of HDACs using HDACis (**Table 3**). The first HDACi discovered was trichostatin A, a dienohydroxamic acid obtained from *Streptomyces* that effectively suppressed zinc-dependent HDACs in the preclinical stage (111). Notably, numerous pan-HDAC inhibitors (P-HDACi) such as vorinostat (also known as suberoylanilide hydroxamic acid, SAHA) (112), belinostat (PXD-101) (113), panobinostat (LBH589) (114), pracinostat (MEI Pharma) (115), romidepsin (FK228) (116), and chidamide (CS055, HBI-8000) (117), have been approved by the FDA to treat different cancers, including primary cutaneous T-cell lymphoma, peripheral T-cell lymphoma, MM, and AML. Moreover, several P-HDACis, including resminostat (4SC-201) (118), givinostat (ITF2357) (119), quisinostat (JNJ-26481585) (120), entinostat (121), and mocetinostat (122), have been evaluated clinically for Hodgkin’s lymphoma, polycythemia, ovarian cancer, and other carcinomas. Furthermore, both sirtinol and nicotinamide have exhibited activity against breast cancer as SIRT inhibitors (123, 125) (**Table 3**). In addition to the compounds mentioned, novel HDACis are constantly being developed (25).

### 4.4 Inhibitors Targeting Specific Functional Domains of Acetyl-Lysine Readers

Acetyl-lysine on histones can also be recognized by readers with specific functional domains, such as PHD finger, Yaf9, ENL, AF9, Taf14, and Sas5 (YEATS), and BRD. PHD finger proteins recognize acetylated, un-acetylated, or methylated histones, with the PHD finger domains in MLL4 (KMT2D) and MLL3 (KMT2C) targeting H4K16 acetylation and involved in the interaction between MLL4/3 and males absent on the first (MOF) (1278). YEATS proteins interact with acetylated histones H3K9, H3K14, and H3K27 (126).

Many BRD proteins are involved in chromatin-remodeling or chromatin-modifying enzymes. BRDs in HATs act as protein–protein interaction modules that specifically recognize acetylated histones to regulate gene transcription, including H4K5, H4K8, H4K12, H4K16, H4K20, H3K14, and H3K36 (5). BRD proteins are the most widely studied acetyl-lysine readers and have been found in many nuclear proteins, including HATs, HMTs, chromatin-remodeling enzymes, and transcriptional co-activators (127). At present, several inhibitors that target the acetyl-binding pocket of BRDs or BRD extraterminal proteins (BETs) have been discovered (25) (**Table 4**). Among them, BET inhibitors such as RVX-208 (RVX00022), I-BET762 (GSK525762), FT-1101, CPI-0610, BAY1238094, INCB054329, PLX51107, GSK2820151, ZEN003694, BMS-986158, BI 894999, ABBV-075, GS-5829 (128, 129), and OTX015 (MK-8628) have been tested for their anti-tumor effects against numerous types of cancers in clinical trials (130, 131) (**Table 4**). Moreover, several novel BRD inhibitors, including I-BRD9, BI-7273, and BI-9564, can specifically target BRD9 and possess anti-tumor activity (132, 133).

**Table 4:** BRD-extraterminal proteins inhibitors (BETi) display the roles against tumors.

Compound	Tumor types	Status	Ref.
GSK525762/I-BET762	Breast Cancer	Phase I	(128)
FT-1101	AML	Phase I	(128)
CPI-0610	MM	Phase I	(128)
BAY1238094	N/A	N/A	(128)
INCB054329	Solid Tumors	Phase I/II	(128)
PLX51107/GSK2820151	Solid Tumors	Phase I	(129)
ZEN003694	Prostate Cancer	Phase I	(129)
BMS-986158/GS-5829	Solid Tumor	Phase I	(129)
BI 894999	Neoplasms	Phase I	(129)
ABBV-075	Breast Cancer	Phase I	(129)
MK-8628/OTX015	AML	Phase I	(130)

## 5 Epigenetic Enzymes as Anticancer Targets of Natural Compounds and Their Active Components

**Figure 3:**
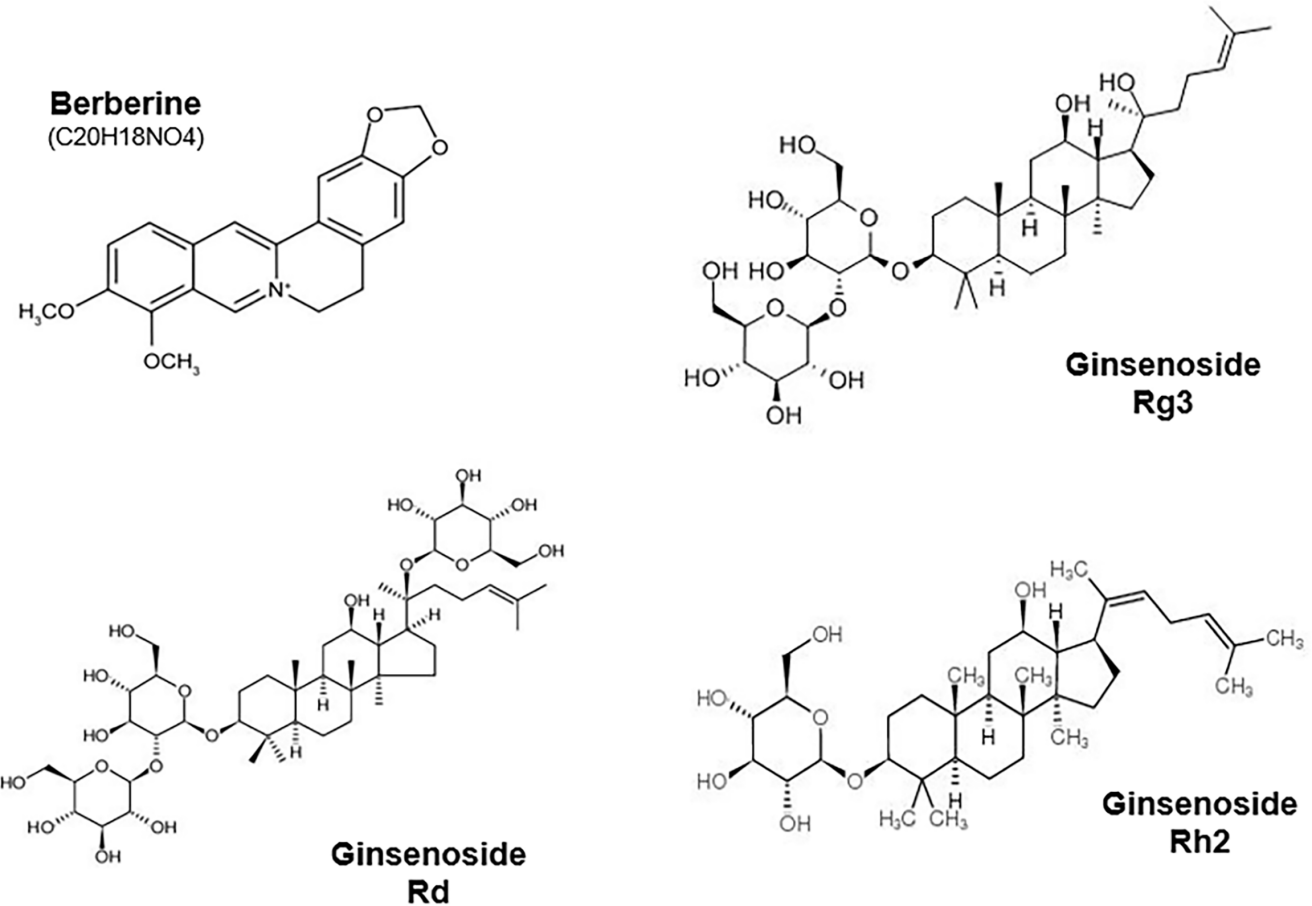
Chemical structure of berberine and Ginsenosides Rh2, Rg3 and Rd.

Natural compounds and their active components have been widely used in traditional medicine in China, Japan, South Korea, and other countries for their various pharmacological effects. Increasing natural compounds have demonstrated high anticancer activity, providing potential candidates for developing multifunctional tumor-targeted drugs. However, their precise mechanisms of action remain unclear. Here, we focus on BBR (C20H18NO4) and ginsenosides, natural compounds that have undergone extensive preclinical investigation and play anti-tumor roles by targeting epigenetic enzymes and ncRNAs (**Figure 3**).

### 5.1 Berberine, a Natural Compound With Epigenetic Regulatory Activity

BBR, the main alkaloid in the herbal medicine *Coptis*, and its derivatives exhibit effective anti-tumor activity (**Table 5**). The functional mechanism of BBR is closely related to its regulation of epigenetic chromatin-modifying enzymes, as the activities of multiple enzymes involved in histone acetylation and methylation, such as CBP/p300, SIRT3, KDM6A, SETD7, and HDAC8, are altered when myeloma U266 cells are treated with BBR (138). Furthermore, BBR treatment leads to the increased acetylation of histones H3 and H4 and suppresses total HDAC activity, further retarding the growth of human lung cancer A549 cells (139). Chen et al. demonstrated that BBR reduces the expression of both EZH2 and H3K27me3 in esophageal carcinoma (134). Further, pseudodehydrocorydaline (a protoberberine alkaloid) selectively suppresses the activity of HMT G9a and decreases the expression of H3K9me2 in MCF-7 breast cancer cells *via* CT13 occupation of the binding site of histone H3, suggesting that CT13 might provide a novel scaffold for synthetic G9a inhibitors (135). In addition to modifying histones, BBR also regulates DNMTs. BBR reportedly accesses chromatin in hepatoma HepG2 cells, resulting in increased global genome methylation and reduced methylation in promoter region CpG sites of cytochrome P450 2B6 (*CYP2B6*), cytochrome P450 3A4 (*CYP3A4*), and glucose regulated protein 78 (*GRP78*) (136). In addition, BBR effectively reduces the expression of DNMT1/3B and promotes p53-hypomethylation, thus further altering the p53-dependent signaling pathway to hinder the growth of myeloma U266 cells (137, 140).

**Table 5:** Berberine targets epigenetic enzymes for cancer therapeutics.

Compound	Targets	Tumor types	Ref.
Berberine	EZH2/H3K27me3	Osteosarcoma	(134)
Berberine	Global histone H3/H4 acetylation	Lung cancer	(135)
Berberine	DNMTs	Gastric cancer	(136)
Berberine	DNMT1/DNMT3B	MM	(137)

### 5.2 Anti-Tumor Epigenetic Regulatory Effects of Ginsenosides

Ginsenosides, derived from saponins of ginseng, have a steroid-like hydrophobic backbone connected to one or more sugar moieties and are generally believed to be the major bioactive constituents of ginseng (141). Ginsenosides are divided into two groups based on their chemical structures, panaxatriol (Re, Rf, Rg1, Rg2, and Rh1) and panaxadiol (Rb1, Rb2, Rb3, Rc, Rj, Rg3, and Rh2) (142) (**Table 6**). Although ginsenosides possess various pharmacological activities, including anti-inflammatory, anti-allergic, anti-fatigue, anti-stress, and anti-cancer properties (152), their basic biological characteristics have not been fully studied. Recent studies have demonstrated that epigenetic mechanisms might be involved in pharmacological effects of ginsenosides (153).

**Table 6:** Ginsenosides target epigenetic enzymes against cancers.

Compound	Targets	Tumor types	Ref.
Ginsenosides Rh2	Hyper-methylated genes	Breast cancer	(143)
Ginsenosides CK	DNMT1	Colorectal cancer	(144)
Ginsenosides Rg3	DNMTs	Ovarian cancer	(145)
20(S)-ginsenoside Rh2	HDACs	Leukemia	(146)
Ginsenosides Rh2	MiR-222/MiR-34a/MiR-29a	Breast cancer	(147)
20(S)-ginsenoside Rg3	MiR-145	Ovarian cancer	(148)
Ginsenosides Rg3	MiR-221	Oral squamous carcinoma	(149)
Ginsenosides Rh2	IncRNA C3orf67	Breast cancer	(150)
Ginsenosides Rg3	IncRNA RFX-AS1/STXBP5-AS1	Breast cancer	(151)

Genome-wide DNA methylation analysis revealed that ginsenoside Rh2 inhibits the growth of breast cancer MCF-7 cells by reducing long interspersed nucleotide element methylation and the expression of hypermethylated genes involved in tumorigenesis (143). Similarly, ginsenoside Rg3 treatment downregulates hypermethylated tRNA methyltransferase 1-like (TRMT1L), proteasome 26S subunit, ATPase 6 (PSMC6), and NADPH oxidase 4 (NOX4), while upregulating hypomethylated ST3 beta-galactoside alpha-2, 3-sialyltransferase 4 (ST3GAL4), RNLS, and KDM5A in breast cancer MCF-7 cells to block tumor growth (154). Ginsenosides also block DNMTs by modulating their target genes. Compound K (the main metabolite of ginseng saponin) suppresses *DNMT1* expression to reduce the proliferation of colorectal cancer (CRC) cells by reactivating the epigenetically silenced *RUNX3* gene (144). Ginsenoside Rg3 treatment decreases the expression of DNMT1/3A/3B and increases the acetylation of histones H3K9/K14 and H4K5/K12/K16 to inhibit the growth of ovarian cancer cells (145). Treatment with 20(s)-ginsenoside Rh2 suppresses the proliferation of K562 and KG1-α leukemia cells by reducing the expression and activity of HDACs, including HDAC1/C2/C6, suggesting that 20(s)-ginsenoside Rh2 acts as an HDACi (155). Interestingly, treatment with ginsenoside Rh2 also suppresses PDZ-binding kinase/T-LAK cell-originated protein kinase (PBK/TOPK), which retards the proliferation of tumor cells through the ERK1/2 signaling pathway (156).

A recent study reported the ability of ginsenosides to suppress cancer by regulating miRNAs (150, 157). The activity of ginsenoside Rh2 against different types of cancer cells is mediated by upregulating miR-146a-5p, miR-21, miR-491, and miR128 (158–160) or by downregulating miR-4295, miR-31, and miR-638 (146, 161–163). In addition, treatment with the ginsenoside Rh2 reduces anti-tumor drug resistance in breast cancer cells by reducing the expression of miR-222, miR-34a, and miR-29a (147). Further, treatment with 20(S)-ginsenoside Rg3 reverses epithelial–mesenchymal transition in ovarian cancer cells by downregulating DNMT3A-mediated miR-145 (148). Similarly, ginsenoside Rg3 treatment downregulates miR-221 to reduce epithelial–mesenchymal transition in human oral squamous carcinoma cells (149). 20(S)-ginsenoside Rg3-mediated miR-532-3p/miR-324-5p also represses the expression of pyruvate kinase M2 (PMK2), resulting in an anti-tumor effect (164, 165).

Ginsenosides also modulate lncRNAs to hamper the growth of cancer cells (150, 166). Treatment with ginsenoside Rh2 suppresses the lncRNA C3orf67 in breast cancer MCF-7 cells (151). Moreover, ginsenoside Rg3 binds to the promoters of two lncRNAs, regulatory factor X-antisense 1 (RFX-AS1) and syntaxin-binding protein 5-antisense 1 (STXBP5-AS1), to alter DNA methylation, thus inhibiting the growth of breast cancer MCF-7 cells (56) (**Table 6**). Thus, ginsenosides mediate the expression of DNMTs and lncRNAs in tumor growth. In summary, natural compounds and their active components, targeting epigenetic enzymes, have therapeutic potential for cancer treatment (57).

## 6 Conclusions and Perspectives

Epigenomic alterations mediated by epigenetic regulatory enzymes have a profound effect on many hallmarks of cancer, including malignant self-renewal, differentiation blockade, evasion of cell death, and tissue invasiveness (167). The anticancer roles of inhibitors targeting epigenetic regulatory enzymes provide attractive targets for novel drugs, even if enzymes that selectively regulate the target genes are not well known (168). Some HATis, HDACis, and DNMTis have been approved as anticancer epidrugs. However, the use of most epigenetic regulatory enzyme inhibitors is limited by their poor bioavailability, cytotoxicity, and specificity. Therefore, developing effective drugs that target epigenetic enzymes remains challenging. An increasing number of studies has demonstrated that many natural compounds and their active components target epigenetic enzymes to successfully delay cancer progression, suggesting attractive alternatives for anticancer treatments.

## Author Contributions

YJ, TL, HL, YL, and DL participated in writing, editing, and creating figures. All authors have read and approved the final manuscript.

## Funding

This research was funded by the National Natural Science Foundation of China (Grant Nos. 82003985 and 81973712). The Science and Technology Research Project of the Jilin Provincial Department of Education (Grant No. JJKH20210995KJ, JJKH20210995KJ), and Jilin Province Science and Technology Development Project in China (Grant No. 20210204013YY).

## Conflict of Interest

The authors declare that the research was conducted in the absence of any commercial or financial relationships that could be construed as a potential conflict of interest.

## Publisher’s Note

All claims expressed in this article are solely those of the authors and do not necessarily represent those of their affiliated organizations, or those of the publisher, the editors and the reviewers. Any product that may be evaluated in this article, or claim that may be made by its manufacturer, is not guaranteed or endorsed by the publisher.

## Abbreviations

AML, acute myeloid leukemia; BBR, berberine; BET, BRD-extra terminal protein; BRD, bromodomain; CHD, chromo-ATPase/helicase-DNA-binding; CDY, chromodomain Y; CpGI, CpG island; CRC, colorectal cancer; DNMT, DNA methylase; DNMTi, DNMT inhibitor; DOT1, disruptor of telomeric silencing 1; DOT1L, DOT1-like; EZH1/2, enhancer of zeste homologs 1/2; FAD, flavin adenine dinucleotide; GNAT, GCN5-related N-acetyltransferase; HAT, histone acetyltransferase; HDAC, histone deacetylase; HMT, histone methyltransferase; HMTi, HMT inhibitor; HP1, heterochromatin protein 1; JmjC, Jumonji C; KDM, lysine demethylase; KMT, lysine methyltransferase; LSD, lysine-specific demethylase; MBD, methyl-CpG-binding domain; MBP, methyl-CpG-binding protein; MBT, malignant brain tumor; mC; miRNA, microRNA; MLL, mixed lineage leukemia; MM, multiple myeloma; MTase, methyltransferase; MYST, MOZ, YBF2/SAS3, SAS2, and TIP60; NA, nucleoside analog; NB, neuroblastoma; ncRNA, noncoding RNA; NHL, non-Hodgkin’s lymphoma; PHD, plant homeodomain; PRMT, protein arginine methyltransferase; PRMTi, PRMT inhibitor; PWWP, Pro-Trp-Trp-Pro; SAM, S-adenosylmethionine; SCLC, small cell lung cancer; SET, su(var) 3-9, enhancer of zeste, and trithorax; SIRT, sirtuin; TET, ten-eleven translocation.
